# Development of a web-based patient decision aid for myopia laser correction method

**DOI:** 10.1186/s12911-024-02559-3

**Published:** 2024-06-05

**Authors:** Hanieh Delshad Aghdam, Fatemeh Zarei, Seyed Farzad Mohammadi

**Affiliations:** 1 Department of Health Education and Health Promotion, Faculty of Medical Sciences, Tarbiat Modares University, Tehran Jalal AleAhmad Nasr, P.O.Box: 14115-111, Teharn, Iran; 2 Translational Ophthalmology Research Center, Farabi Eye Hospital, Tehran University of Medical Sciences, Tehran, Iran

**Keywords:** Patient decision aid, Myopia, Laser correction

## Abstract

**Background:**

In the context of healthcare centered on the patient, Patient Decision Aids (PtDAs) acts as an essential instrument, promoting shared decision-making (SDM). Considering the prevalent occurrence of myopia, the objective of this study is to furnish exhaustive and easily comprehensible information to assist patients in making well-informed decisions about their options for myopia laser correction.

**Method:**

The research team developed a decision guide for myopia patients considering laser correction, aiming to facilitate informed decisions. The study followed the first four stages of the IPDAS process model: “scope/scoping,” “design,” “prototype development,” and “alpha testing.” Ten semi-structured interviews with patients (*n* = 6) and corneal specialist ophthalmologists (*n* = 4) were conducted to understand the challenges in selecting a laser correction method. Online meetings with 4 corneal specialists were held to discuss challenging cases. A comparison table of harms and benefits was created. The initial prototype was developed and uploaded on the internet portal. User feedback on software and text aspects was incorporated into the final web software, which was reviewed by a health education expert for user-friendliness and effectiveness.

**Result:**

Educational needs assessment revealed concerns such as pain, daily life activities, return to work, the potential need for glasses (‘number return’), eye prescription stability, and possible complications. These shaped the decision aid tool’s content. Expert consensus was achieved in several areas, with some items added or extended. In areas lacking consensus, comments were added for clarity. Five clients assessed the web app (PDAIN), rating it 46/50 in user-centricity, 47/50 in usability, and 45/50 in accuracy and reliability, totaling 138/150. Post-piloting, software errors were documented and rectified. During the trial phase, five myopic users interacted with the software, leading to modifications. User feedback indicated the tool effectively enhanced understanding and influenced decision-making.

**Conclusion:**

PDAIN, serves as a facilitative tool in the process of selecting a corneal laser correction method for myopic patients. It enabling Nearsighted patients to make informed decisions.

**Supplementary information:**

The online version contains supplementary material available at 10.1186/s12911-024-02559-3.

## Background

Patient-centered care is a healthcare approach in which the patient is positioned at the core of their care. This concept, which has been increasingly emphasized in contemporary medical discourse, underscores the critical importance of individualized care in promoting optimal health outcomes [1]. 

Shared decision-making constitutes a fundamental component of patient-centered care in the realm of healthcare practices [2, 3]. Clinical guidelines advocate for the active participation of patients in decision-making processes about screening, treatment, and therapeutic interventions, to facilitate informed decision-making. Patient Decision Aid (PDA) tools are a supplement in this process and not a substitute for expert-patient interaction [4, 5]. Such tools are employed in healthcare scenarios where multiple treatment strategies, methodologies, or options are available, and where it is ethically permissible for the individual to not choose from one of two or more [6]. These instruments are specifically designed to address a particular problem or topic, underscoring the notion of decision-making as a procedural endeavor [4, 7, 8].These instruments are distinguished from patient education tools. Beyond merely disseminating information, they assist individuals in recognizing their values and preferences within the decision-making process. This recognition may subsequently drive a need for additional information or a reconsideration of the decision at hand [6].

PDAs furnish information regarding the spectrum of available treatment options, thereby facilitating patients in making decisions that align with their values. Empirical studies have demonstrated that PDAs enhance patients’ comprehension of the available treatment alternatives, their understanding of associated risk factors, and their recognition of personal values in the decision-making trajectory, thereby fostering improved patient-doctor communication. At the University of Ottawa, a decision-making instrument tailored for patients with myopia has been developed, adhering to the standards of these tools, and is accessible online [9]. 

### Global trends and myopia prevalence

Refractive errors represent a prevalent ocular disorder across all age demographics, posing a significant public health challenge [10, 11]. The incidence of myopia, or nearsightedness, varies significantly across the world. It ranges from a low of 0.8% among children aged 6–11 in Laos to a high of 86.5% among individuals aged 15–19 in China, demonstrating a wide spectrum of prevalence rates. In 2010, the number of individuals with myopia in Europe was reported to be 227.2 million. Notably, an upward trend in the prevalence of myopia has been observed in East Asian countries [10, 11]. Projections indicate that by the year 2050, the prevalence of myopia and high myopia will escalate to 52% (equivalent to 4949 million individuals) and 10% (equivalent to 925 million individuals), respectively [12, 13]. In Iran, the prevalence of myopia fluctuates between 4% and 30% across different cities. Notably, the incidence is higher among individuals aged 15–35 and 45–55 years compared to other age groups. The elevated prevalence of high myopia in the 45–55-year age bracket is associated with the onset of cataracts during this period. However, studies investigating the prevalence of this condition in Iran, and specifically Tehran, remain limited [14, 15].

The principal risk factors implicated in the onset of refractive eye errors encompass the age [15, 16] of the individual and the duration spent outside enclosed environments (such as homes, workplaces, schools, etc.), genetic predisposition, and a family history of this disorder [12]. Currently, there are no established methods for treating or preventing this condition; only strategies to correct it or suspend its progression are available. Over the past decades, various approaches to control the progression of myopia have been evaluated. These include the use of under-corrected glasses (with lower numbers), bifocal glasses, and other types of corrective methods, hard, bifocal, and peripheral focus contact lenses, orthokeratology, and others [12]. 

Refractive surgeries in the recent decade have progressed and nowadays there are many different types of surgeries, mainly including Photorefractive Keratectomy (PRK), Laser Epithelial Keratomileusis (LASEK), Laser in Situ Keratomileusis (LASIK), and Small Incision Lenticule Extraction (SMILE) [17]. In some cases, the patient could make a selection among SMILE, Femto-LASIK, and PRK. Myopia is the primary cause of visual impairments and the secondary cause of vision loss globally [12, 13]. Lifestyle changes and the increasing tendency to engage in activities within enclosed spaces (such as workplaces, schools, and homes) have contributed to a rise in the prevalence of myopia worldwide and the need to select corrective methods is rising [10, 11]. Providing patients with structured information, grounded in the decision-making process, can enhance the quality of informed decisions. Consequently, patients will harbor more accurate and realistic expectations of specific health services and acquire the skill of collaborative decision-making with their healthcare provider [16]. Given the escalating prevalence of myopia, the breadth of common treatment methods, and the confusion patients often face when selecting an appropriate method, it appears both beneficial and necessary to educate these patients via the design and evaluation of decision-making tools.

**Fig. 1:**
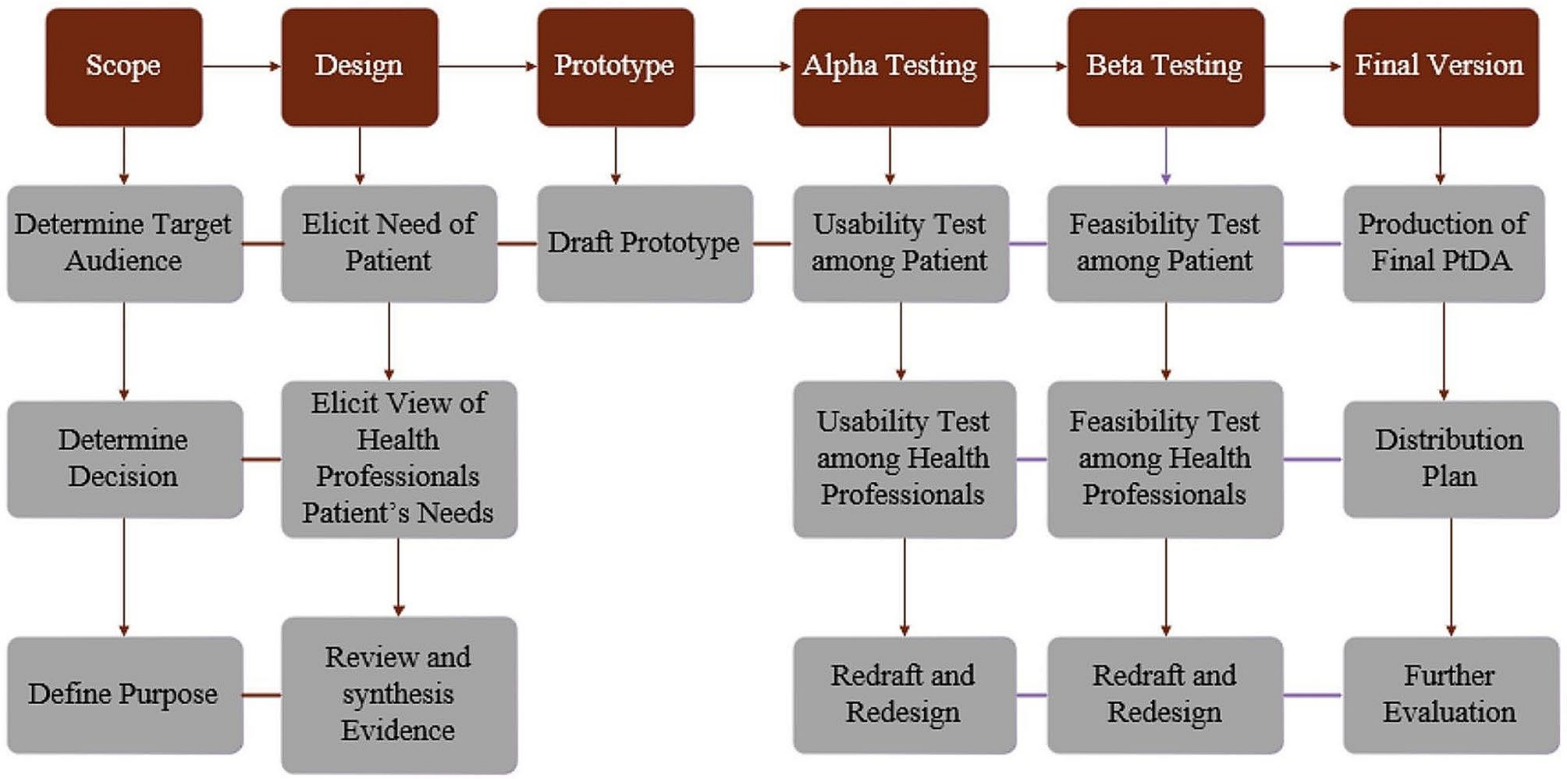
Systematic development process of PtDA according to IPDAS

The International Patient Decision Aid Standards Group (IPDAS) comprises a global consortium of researchers, clinicians, and stakeholders. Established in 2003, the group embarked on a mission to enhance the quality and efficacy of patient decision aids. To this end, they developed an evidence-based framework encompassing a set of criteria aimed at improving various aspects such as content, development, implementation, and evaluation [18]. IPDAS advocates for a meticulous approach to the development of Patient Decision Aids (PtDAs). This involves careful construction, user testing, and rigorous review of the aids. Furthermore, IPDAS emphasizes the importance of documenting the development process systematically to ensure transparency and reproducibility. This rigorous approach is aimed at enhancing the quality and effectiveness of PtDAs [19]. The process model proposed by IPDAS delineates a comprehensive and systematic methodology for the development of PtDAs that are subject to user testing and review (Fig. 1) [19]. 

## Method

The current study concentrates on the initial four stages of the IPDAS process model, namely: “scope/scoping”, “design”, “prototype development”, and “alpha testing”. This process was executed by a multidisciplinary team composed of a corneal specialist (ophthalmologist), an ocular nurse, a specialist in health education and promotion, and a seasoned web designer. This collaborative approach aimed to ensure a comprehensive and effective development process for (PtDAs).

### Scope

Given the widespread incidence of myopia, the diversity of refractive laser surgeries, and the specific needs of patients, the research team intends to develop a decision guide tailored to the laser correction methodology for patients with myopia. This initiative aims to provide comprehensive and accessible information to aid patients in making informed decisions regarding their myopia laser correction options.

### Design

#### Need assessment

The research team utilized interviews with both patients and ophthalmologists to ascertain the requirements and challenges associated with selecting a laser correction method for myopia. In semi-structured 10 interviews, myopic patients were queried about the difficulties they encountered when deciding on an appropriate laser correction method for myopia. The question posed was: “Given that you are about to undergo laser eye correction surgery, what factors could assist you in deciding among the three available corneal laser methods? What could facilitate your decision-making and choice of method?” Concurrently, ophthalmologists were interviewed regarding the challenges they faced during counseling sessions and decision-making processes with these patients. They were asked: “In the context of counseling for the selection among three surgical methods (SMILE, Femto-LASIK, and PRK), what challenges have you encountered with patients in reaching a decision and making a choice?” This comprehensive approach aimed to gain a holistic understanding of the decision-making process from both the patient and practitioner perspectives. In this phase of the study, a targeted sampling method was employed and continued until data saturation was achieved. After implementation, the data was subjected to a three-round review and thematic analysis using MAXQDA software version 22. Given the novelty of the subject matter, and the scarcity of sufficient clinical evidence and sample software in the desired field, a comprehensive search was conducted. Consequently, the classic expert consensus method was selected to compare challenging cases in patients’ decision-making across three methods. Four online meetings were convened with distinguished professors of ophthalmology and a cornea specialist. During these meetings, the initial text comparing the three surgical methods across different fields, based on the titles extracted from the needs assessment, was reviewed. Each session was attended by an expert in health education and health promotion, an ophthalmologist (corneal specialist), and a guest professor. Each item in the table was discussed and exchanged. Following each session, the audio file was reviewed in the form of comments over the next 1 to 3 days. Subsequently, the table was reviewed and rewritten by the ophthalmologist. In the subsequent meeting, the table was revised and reviewed, and this cycle was repeated four times. Upon the conclusion of the four sessions, a summary was prepared. There were instances where consensus was not reached during these meetings. For such cases, the disagreement was noted in the form of a comment with a clear explanation in the table. Finally, a comparison table of harms and benefits was compiled based on the results (Appendix 1).

### Development of initial prototype

IPDAS outlines five primary components of any standard decision support software:

1. **Get the Facts**: Include information from various sources and expert opinions, and provide detailed explanations about the available choices. An explanation of myopia, a list of choices, an explanation of non-surgical correction methods, a brief explanation of each surgical process, and an explanation of the advantages, disadvantages, and effects of each method are given in this section. These explanations are purposefully compiled without referring to details unrelated to decision-making.
2. **Comparison of Choices**: This section necessitates the presentation of items that aid the patient in making a decision and comparing these items across all three methods. The comparison table of harms and benefits is compiled based on the results of consensus meetings of ophthalmologists. Other parts of the software are compiled based on the extracted comparison table.
3. **Your Feelings**: In this section, questions are formulated for self-evaluation of patients’ preferences in selection using a Likert scale. After receiving the facts and comparing the benefits and harms of each method, patients rate their priorities and gain an understanding of their feelings.
4. **Self-examination**: This involves compiling questions for self-evaluation of acquired knowledge. By answering the proposed questions from the previous section, patients evaluate their readiness in terms of knowledge to make decisions. In the design of the questions, the number of questions about each method was the same and in the form of one positive point and one negative point about each method so as not to influence the decision-making of the patients.
5. **Decision**: In this section, questions are asked to evaluate the level of readiness of the patient to make decisions and their needs for making decisions. If they are ready, the clients specify the surgical method they want.

The compiled materials are arranged and uploaded step by step according to the structure of decision-making tools in the portal (Internet). Without completing the current stage, it is not possible to pass it and return to the previous stages. In the end, a summary of the clients’ choices along with the correct answers of the self-test is provided to them. The researchers of this study have named this tool “Ratal,” a Farsi term derived from the phrase “decision aid guide for choosing the laser correction method for myopic patients.” In English, this tool is referred to as PDAIN, an acronym for “Patient Decision Aid for Iranian Nearsighted.” Since the readers of this article are from diverse linguistic backgrounds, this tool will be introduced as PDAIN to cater to a global audience. This name succinctly encapsulates the tool’s purpose and target demographic. It’s a fitting title for a tool designed to aid myopic patients in Iran in making informed decisions about laser correction methods.

### Alpha test

The tool was evaluated by five patients in terms of:

- **Software User**:

1. User-centricity.
2. Usability: include.
  - Clarity of the user’s location.
  - Maintaining the user’s latest status for the next upload.
  - No dependence on other programs.
  - Ease of user access.
  - The number of clicks necessary to reach the user’s goal.
3. Accuracy and reliability.
  - Reliability (reliability) of a system (equipment) is the probability of correct and fault-free operation of that system during a specified and predetermined period with certain conditions and quality.

- **Text**:

1. Clarity.
2. Simplicity.
3. Relevance.

The contributors to this evaluation included a refractive surgery candidate who is an IT engineer, a surgical candidate who is an employee, a Ph.D. student in health education with a history of laser correction surgery, a surgery candidate who is a psychology student, and a website designer who is a senior executive management expert. Additionally, the software was tested by five real users (individuals who visited the eye clinic and were candidates for corneal laser refractive surgery), and an ophthalmologist reviewed and completed different sections, recording errors and comments.

### Finalizing the designed decision aid tool

After incorporating the received feedback, the final web software was reviewed by a health education expert, and the necessary corrections were made by the education expert and the software web writer. This rigorous process ensured the development of a user-friendly and effective decision-aid tool.

## Result

### Extracted educational needs from need assessment phase

The educational needs identified from the interviews are summarized in Table 1. The most frequently mentioned concerns included pain, resumption of daily life activities, return to work, the potential need to use glasses again (‘number return’), stability of eye prescription, and possible complications. These clues were instrumental in shaping the content of the decision-aid tool.

**Table 1:** Titles extracted from the interviews

Row	Title		F*	Row	Title		F*
1	Pain	pain	8	6	Relative superiority of methods in safety and effectiveness	Burning complication after surgery	1
		Pain during surgery	1			The skill of the surgeon	2
		Postoperative headache	1			Need to retreatment	1
		Duration of postoperative pain	1			refractive error regression	3
2	Patient post-operation care need and Patient Complement to the care	The need for Pain reliever	1			keratoconus after surgery	1
		Avoid light (Photophobia) after surgery	1			Need to reuse glasses	3
		The need to use glasses after surgery	1			coverage of refractive error improvement	1
		The need for Patching after surgery	1			tearing post-surgery	1
3	Sport (limitations and restart)	Time allowed to start exercise	1			Complications	3
		Exercise restriction after surgery	1			Astigmatism can be corrected	2
4	Recovery and time to return to Normal life and work After the operation	Need to rest	2			Possibility of corneal opacity after surgery	1
		Back to everyday life	3	7	Surgical procedure experience from the patient	Duration of surgery	2
		Time allowed to travel	1				
		Time to return to work	3			Pain during surgery	1
5	Course and speed of recovery And He recovered	Time to improve vision after surgery	1			Fear of getting something into the eye during surgery	1
		Long-term effect	3	8	Post-Surgery Dry Eye		1
		Long-term vision quality	1	9	Dependence on sufficient Corneal thickness		1
		Vision recovery time	1	10	Up-to-date surgical procedure		1
		Recovering	4	11	Cost		1

F*: Frequency

### Expert consensus meetings

Consensus was achieved in several areas, including the average comparative intensity of postoperative pain or discomfort, vision improvement (with a 90% improvement rate), home rest time, the average probability of requiring a re-operation/enhancement, and the likelihood of needing under LASIK flap or SMILE cap washing or re-floating. The item “Dependence of the safety (and result) of the operation on the surgeon’s skill” was incorporated into the table based on an expert’s suggestion. Furthermore, elements such as “feeling of pain” and “burning sensation” during the surgical procedure were included under the item “discomfort during the surgical procedure”. In the domain of dry eye, by specifying the timeframe as “the first 6 months post-operatively”, the opinions were harmonized. The consensus was reached on the duration of postoperative pain or discomfort by incorporating the term “moderate”. Based on the expert opinions, sports activities were categorized into three groups according to the varying commencement times post-surgery. Opinions on avoiding fasting post-surgery were diverse and varied, with some experts suggesting two weeks, others 6 weeks, some a year post-surgery, and some after the resolution of dry eye symptoms.

There was no consensus on the appropriate time to use decorative or colored contact lenses post-surgery, with suggested durations ranging from two weeks to six months for LASIK. In terms of recommending the use of sunglasses, experts suggested the term “optional” vs. recommended instead of “not recommended”, given the importance of sunglasses in daily life and their general impact on eye health. Regarding the “Approximate duration of ophthalmic drops application” item, due to the necessity of using artificial tear drops to alleviate dry eye symptoms and the varying intensity and duration of dry eyes among different individuals (sometimes up to a year), the term “treatment” was incorporated into the phrase, and the duration of drop usage was specified.

In the “Ophthalmic drug side effects” item, the case of cataracts was removed due to its rare incidence, per expert suggestions, and the risk of increased eye pressure, due to its greater significance, was specifically mentioned: “The risk of side effects of corticosteroid drops (especially increased eye pressure)”. Possibility and ease of re-operation were also added to the table due to patient concerns about the return of eye error. According to experts’ opinion, the vision blurring section was divided into two sections: “vision blurring during the first week after the operation” and “vision blurring during two weeks to three months after the operation”. Similarly, dry eye was expressed in two sections and time courses: “in the first 6 months” and “after 6 months to a year”. Considering the less rest requirement in SMILE compared to LASIK and the lexical similarity in time (second day after surgery), the term “after the first postoperative visit” was used to express the timing of these cases.

### Evaluation of web app

Five clients assessed the interface of a web app “PDAIN”. In the section about the comparison of choices, none of the clients opted for the “completely simple” choice concerning the readability of the text. The “not easy” option was not selected by users for any of the software sections. In terms of relevance, users predominantly chose the options “completely relevant” and “relevant”. In the clarity section, only one user selected the “relatively clear” option in the “comparing the choices” section, while the remaining users selected the options “quite clear” and “clear” for all sections. In the user evaluation section, the software received a score ranging between 7 and 10 (on a scale from 0 to 10). The lowest score was assigned to the accuracy and reliability section by one user (Table 2).

**Table 2:** Website text evaluation

	readability				Related				Clarity			
	Quite simple	Simple	Relatively simple	Not simple	Totally relevant	Related	Relatively relevant	Not relevant	Quite clear	Clear	Relatively clear	Not clear
Main page	3^*^	0	2	0	4	1	0	0	5	0	0	0
“Receiving the facts”	2	1	2	0	5	0	0	0	4	1	0	0
Selection comparison	0	4	1	0	5	0	0	0	4	0	1	0
Your feelings	3	1	1	0	4	1	0	0	4	1	0	0
Self-test	1	3	1	0	4	1	0	0	4	1	0	0
Your decision	2	2	1	0	3	2	0	0	3	2	0	0

* The Number of reviewers select the items

The total user rate on the website in the three dimensions was: 46 (of 50) in user-centricity, 47 (of 50) in usability, and 45 (of 50) in accuracy and reliability, and a total 138 of 150. Following piloting the software, the errors encountered during the completion of the software were documented and rectified. Given the specialized nature of the tool, vigilant attention was paid to the simplicity of the text. One of the issues encountered with the web user software on iOS devices was resolved through collaboration with a software engineer. This iterative process of evaluation and refinement ensured the development of a user-friendly and effective decision-aid tool.

### Finalization of the tool

During the trial phase, five users with myopic refractive errors interacted with the software. Based on their feedback, the following modifications were made:

1. The explanation of the flap in the ‘Statement of Facts’ section was revised.
2. An error in providing the correct test answer was rectified.
3. A browsing time allowance of 48 h was set, with the provision for the software admin to extend this duration if an extension is requested.

The users’ feedback was instrumental in refining the tool. Some of the comments included:

- “My doubts went down from 90–20%.”
- “These are all 50/50.”
- “It added a lot to my knowledge and influenced my decision.”

These comments indicated that the tool was effective in enhancing users’ understanding and influencing their decision-making process.

## Discussion

This article introduces PDAIN, a web-based decision-making guide software designed to assist myopic patients in choosing a laser correction method (PRK, LASIK, or SMILE). PDAIN is the first Persian Patient Decision Aid (PDA) for this purpose. It was developed to facilitate patient participation in the decision-making process alongside other similar PDAs like EyeChoose. Subbaraman et al. (2022)[20]. developed EyeChoose, a PDA designed to help patients select the most appropriate refractive surgery procedure. Their development process involved a focus group study with participants aged 18–24 years who had nearsightedness, farsightedness, or astigmatism. The researchers aimed to Identify factors influencing patients’ choice of procedure, gather feedback on existing refractive surgery information tools, and Collect requirements for a patient-centered PDA tool(ref). Following the focus group, EyeChoose was designed based on Preferred Practice Pattern (PPP) from the American Academy of Ophthalmology (AAO)[21], the focus group findings, and consultations with ophthalmologists. EyeChoose offers four key features: **Comprehensive Patient Education**: Provides general information about various refractive eye surgery procedures; **Personalized Assessment**: Collects the user’s medical history and preferences regarding factors like procedure benefits, cost, side effects, recovery, and expected outcomes; **Tailored Surgery Recommendations**: Generates personalized recommendations based on the user’s information; and **Local Surgeon Referral**: Connects patients with qualified surgeons in their area. In comparison, PDAIN focuses on assessing patients’ readiness for decision-making. It was developed using an exploratory needs assessment approach and incorporates expertise from ophthalmologists and patients. “PDAIN” has several distinguishing features: **Readiness Assessment**: Measures patients’ level of preparedness to decide on refractive eye surgery; **Context-Based Content**: Utilizes content derived from the needs and perspectives of patients and experts involved in the tool’s development; **“Must-Know” Information**: Focuses on providing essential information for informed decision-making [22]. Both EyeChoose and PDAIN offer valuable tools for informed decision-making about refractive eye surgery. EyeChoose provides comprehensive education, personalized assessments, and surgeon referrals, while PDAIN emphasizes readiness assessment, contextualized content, and essential information. The choice between these tools may depend on individual patient preferences and specific decision-making needs. Patient decision-aid tools empower patients to make informed healthcare choices. EyeChoose and PDAIN are promising examples, each with strengths that can benefit patients undergoing refractive eye surgery. Further research, including larger-scale studies, is warranted to determine the long-term impact of these tools on patient decision-making, satisfaction, and treatment outcomes.

The production results indicated that “PDAIN” has a low error rate and received moderate to high user evaluations, demonstrating its potential to facilitate and enhance decision-making for both patients and their specialists. During the educational needs assessment stage and interviews with ophthalmologists, it became evident that a detailed explanation of the purpose of decision-making software was necessary. Furthermore, the concept of patient participation in decision-making needed to be clarified. As one expert noted, “In my opinion, if the patient trusts his doctor, he will accept his doctor’s decision.” In Nota et al.‘s study, it was highlighted that the acceptance of decision support software by doctors required more attention [23] By expressing the needs of patients in interviews with doctors, this issue was somewhat mitigated. One of the crucial aspects in the development of such software is the engagement of experts. This collaborative approach ensures the creation of a tool that is both effective and user-friendly. In the current research, an initial assessment of patient needs was conducted, followed by interviews with ophthalmologists. The articulation of these needs elicited further perspectives from the ophthalmologists. The interview questions not only explored patient needs but also addressed the needs and challenges faced by specialists during patient consultations. This approach ensures the enhancement of content while mitigating disruptions in the routine workflow of patients. The software’s results can be utilized by the patient in subsequent consultation sessions, enabling more precise discussions with their ophthalmologist if required. The systematic review by LC et al. demonstrated that decision aids across various conditions and decisions can enhance patient knowledge about options, risk comprehension, feelings of being informed, and confidence in their decisions [24]. These findings align with the patient feedback during the trial phase of “PDAIN” on myopic patients. It is noted that the very list of parameters for comparing the laser modalities covers the absolute factors for deciding to do the surgery or not at all; however, the candidates have already made their decision to do the surgery when they come to the eye surgeons. We would like to emphasize that as the tool intends to provide personalized decision-making assistance, we avoided assigning relative weights for the parameters. Like any other application, “PDAIN” too needs periodic updates.

### Limitations and future research

A key limitation of this study is the relatively small sample size. This limited our ability to analyze data by subgroups, such as age groups. However, this initial pilot study served a crucial purpose. By focusing on a smaller group of cornea specialists and health educators, we were able to gather valuable feedback on the usability, functionality, and educational impact of the “PDAIN” tool. This feedback is essential for further development and refinement. The next logical step is a larger-scale randomized controlled trial (RCT) to definitively assess PDAIN’s effectiveness. This would involve comparing patient knowledge and decision-making between a group using “PDAIN” and a control group receiving standard educational materials. Additionally, recruiting a larger and more diverse sample with a wider age range would allow us to explore potential age-related variations in the tool’s effectiveness. The learnings from this pilot study will guide future refinements of PDAIN, maximizing its impact on myopic patients across various demographics.

## Conclusion

This study demonstrates the promise of “PDAIN” as a patient decision-aid tool for myopic patients considering corneal laser correction surgery. The tool empowers patients with knowledge, fostering informed decision-making. For ophthalmologists, “PDAIN” can streamline the preoperative counseling process, particularly for younger patients who often have some level of pre-existing information. By reducing the time needed to explain surgical options, “PDAIN” allows for more focused and efficient consultations, ultimately benefiting both patients and specialists. Future research with larger and more diverse populations will further solidify the role of “PDAIN” in improving patient education and decision-making in refractive surgery.

### Electronic supplementary material

Below is the link to the electronic supplementary material.

Supplementary Material 1

## Data Availability

Because the data includes many indirect patient identifiers, the data of this study is not publicly available. The datasets used and/or analysed during the current study available from the corresponding author on reasonable request.

## Abbreviations

IPDAS: International Patient Decision Aids Standards
PsA: Psoriatic Arthritis
PtDA: Patient Decision Aid; RA: Rheumatoid Arthritis
SDM: Shared Decision
