# Supplementary material for: Development of a web-based patient decision aid for myopia laser correction method
Source: BMC Med Inform Decis Mak. 2024 Jun 5;24:156. doi: 10.1186/s12911-024-02559-3 (PMC11151511; doi:10.1186/s12911-024-02559-3)
Supplement: Supplementary file 1 — Supplementary Material 1 [file 12911_2024_2559_MOESM1_ESM.docx]

**Appendix 1: Comparison table of laser corneal surgery choices in myopia correction**

| *For the Candidate of all three: PRK, Femto-LASIK, and Smile  ** The desired method should be available in the place where they intend to have surgery | | | | | |
| --- | --- | --- | --- | --- | --- |
| **Row** | **Title** | **PRK** | **Femto-LASIK** | **SMILE** | ***Note** |
| **The patient's experience of the surgical procedure** | | | | | |
| 1-1 | Duration of surgery (two eyes) | Less than 10 minutes | 15 to 20 minutes | 10 to 15 minutes |  |
| 2-1 | Unpleasant feeling of the surgical procedure for the patient on a scale of 0 to 10 (10 is the most unpleasant) | 3 | 6 | 6 | Regardless of the procedure, if the surgeon talks to the patient about the surgical procedure before or during the procedure, the experience will be less unpleasant. |
| **The relative superiority of the methods in safety and effectiveness (permanent/long-term risks after the surgery and additional interventions**  **{re-operation and correction of complications})** | | | | | |
| 1-2 | The accuracy of correcting the astigmatic component of the refractive error on a scale of 0 to 10 (the most accurate) | 8 | 9 | 7 |  |
| 2-2 | Moderate risk of keratoconus after surgery | 1 in 2000 | 1 in 500-1000 | 1 in 1500 | Weakening of corneal tissue strength is more in femto-LASIK (due to the creation of a flap), (for this reason, the choice of femto-LASIK depends on the thickness of the cornea more than the other two methods). |
| 3-2 | Risk of postoperative infection (in the first days) | 1 in 2000 | 1 in 5000 | 1 in 8000 | Observance of personal hygiene plays a key role in preventing this complication. |
| 4-2 | Dependence of the safety (and outcome) of the surgery on the surgeon skill, on a scale of 0 to 10 (10 is the most dangerous) | 3 (less relevant) | 5 (average) | 7 (most) |  |
| 5-2 | Complications related to making a pocket or flap in the corneal tissue and removing the lens from inside the pocket | Not relevant | Low | Low |  |
| 6-2 | The possibility of needing to wash the wound, correct flap wrinkles or remove misplaced covering cells under the flap or into the tunnel. | Not relevant | Low | Very low |  |
| 7-2 | Moderate risk of significant corneal opacity/ulceration | 1 in 500 ^*^ | Very low | Low | This case is one of the side effects of LASEK |
| 8-2 | The risk of side effects of corticosteroid drops (especially increased eye pressure) | +++ | + | + |  |
| 9-2 | Moderate probability of needing re surgery | less than 6% | less than 4% | less than 5% | It varies according to the refractive error |
| 10-2 | Reproducibility and ease of re- surgery | It is possible that the experience of the previous surgery will be repeated for the patient. | It is possible and the bed can be lasered again by lifting the flap of the previous operation. However, the adhesion of the flap sometimes makes it difficult to remove and there is a risk of displacing cells under the flap. | It is not possible, but it is usually possible to perform LASEK or convert the previous smile bed to LASIK. In both cases, the benefits of SMILE are practically eliminated. | In all cases, the cornea must have sufficient thickness to correct the remaining or returned score. |
| 11-2 | The possibility of lifting or lagging the flap as a result of impact during life and restrictions for sports such as wrestling, martial arts, and boxing where the eye is exposed to direct contact. | Not relevant | It is possible  (preferred to avoid this practice) * | Not relevant | In some countries, based on this risk, even though it is small, they do not do Femto-LASIK in the military. Although Femto-LASIK is the most common of these 3 procedures worldwide, the general opinion of surgeons is that it involves more manipulation of the eye. |
| 12-2 | The accuracy and possibility of performing customized corneal cutting combined with laser cutting for refractive error of the eye, in case of irregularity in the surface of the cornea. | It is the best method. | It has average accuracy. | Currently, it is not possible. |  |
| **Postoperative pain** | | | | | |
| 1-3 | Average intensity of post-operative pain or discomfort on a scale of 0 to 10 (most severe) | 5^*^ | 1 to 2 | 1 | *In LASAK, sometimes the intensity of the pain makes a person regret the surgery and it is mentioned as the most severe in life (such as natural childbirth or kidney stone pain). |
| 2-3 | Average duration (hours) of postoperative pain or discomfort | Sometimes up to 72 hours | 4 to 6 hours | 2 to 4 hours |  |
| **The speed of recovery** | | | | | |
| 1-4 | Blurred vision (during the first week after the surgery on a scale of 1 to 10 (10 is the most blurred) | 8 | 1 | 2 |  |
| 2-4 | Blurred vision (during two weeks to three months after the surgery on a scale of 1 to 10 (10 is equivalent to the most blurry) | Medium, 5 | From one month onwards, 0 | From one month onwards, 0 |  |
| 3-4 | Reaching 90% of the final vision and vision improvement | 2 to 4 months | Maximum one week | 10 days to two weeks |  |
| **Dry eyes** | | | | | |
| 1-5 | The possibility of dry eyes and discomfort in the eyes (in the first 6 months) | +++ | +++ | + * | *Smile causes less damage to the surface nerves of the cornea and has a lower chance of dry eyes. |
| 2-5 | Probability of long-term dry eye (after 6 months to a year) | + | ++ | -/+ |  |
| **Recovery and time to return to normal life and work after surgery** | | | | | |
| 1-6 | Bathing allowed | 6th day (after lens removal on the 5^th^ day on average) * | 24 to 48 hours after the surgery | After the first postoperative visit | *From the third day, you can wash your head and body avoiding getting your face wet and splashing water on your eyes, although some surgeons postpone it until removing the lens. |
| 2-6 | Rest time at home and time allowed to go to parties | Afterremoving the lens | The second day after the surgery | After the first postoperative visit |  |
| 3-6 | Appropriate/permissible time to start work | The sixth day after surgery and after removing the bondage contact lens | The second day after the surgery | After the first postoperative visit | Assuming normal recovery |
| 4-6 | Travel | The sixth day after surgery and after removing the dressing lens | The second day after the surgery | After the first postoperative visit | Assuming normal recovery |
| 5-6 | Time to start driving | Average, 10 days after the surgery (at night, later and up to a month) | The second day after the surgery | After the first postoperative visit | and checking the adequacy of vision |
| 6-6 | Eyelid makeup | 2 to 3 weeks later | One week later | 2-3 days later |  |
| 6-7 | When allowed to wear cosmetic/colored lenses | 3 months after the surgery | 1.5 months after the surgery | 3 weeks after the surgery | Some colleagues believe in stricter restrictions. It is also reminded that in all three of these practices and the future, it is possible to use contact lenses to correct the remaining score. |
| **Exercise (restrictions and restarts)**  ***Eye sensitivity to direct impact and indirect shock (such as parading and jumping or diving in water) in patients with a high eye score remains after the surgery and is not related to the type of surgery.** | | | | | |
| 1-7 | Walking, gentle running, and club sports | 1 week | The second day after the surgery | After the first postoperative visit | It is following the bathing permit. |
| 2-7 | types of exercise; Martial arts, wrestling, and boxing | no problem | *There is a small chance of flap lifting and lagging due to trauma to the eye during life. | no problem | *About football (and heading), there is a difference of opinion. |
| 3-7 | Exercise start time: martial arts, wrestling and boxing | 3 weeks | It is prohibited | A week |  |
| 4-7 | Swimming pool (provided wearing suitable swimming goggles) | 3 weeks | 2 weeks | 1 week |  |
| 5-7 | Climbing and other sports | 2 to 3 weeks | 1 to 2 weeks | The third day after the surgery | Some experts postpone any breathing exercise until 4 weeks after the surgery, especially in Lasak, where wound healing takes weeks. |
| **The need for care and the patient's obligations to them after the surgery** | | | | | |
| 1-8 | Having a dressing lens | *Usually, 4 to 6 days | If used, 1 day | It is not usual, but if it is used, 1 day | *Sometimes, due to the delay in recovery or recurrence of the wound, it may be necessary to re-insert the lens and stay longer. |
| 2-8 | Approximate/average duration of instillation of anti-inflammatory drops | *2 to 4 months | 10 days to 2 weeks | 10 days to 2 weeks | Basically, LASAK has a longer medication (eye-drop) and requires more patient adherence. |
| 3-8 | Recommend wearing sunglasses outdoors due to the surgery | 3 to 6 months after the surgery | It is optional | It is optional | In general, the use of sunglasses is necessary to maintain eye health. |
| 4-8 | The need or necessity to use sunglasses at home (for pain relief and protection) | *Much | It is important for trauma care. | It is not necessary | For pain relief and care for shock and fluid splashes |
| 5-8 | Avoid fasting  (in hot weather and long days) | 6 weeks later | 2 weeks later | 10 days later | There are different opinions of experts; Some surgeons forbid it in the coming year, and some compensate for the dryness during fasting with more artificial tears. |
| **Cost** | | | | | |
| 1-9 | Operation cost (two eyes)  scale 0 to 10 | 5 | 8 | 10 |  |
